# A signal sequence multi-leucine motif regulates VEGF receptor signaling

**DOI:** 10.1016/j.jbc.2026.113406

**Published:** 2026-08-07

**Authors:** Mean Ghim, Linyan Wei, Jae-Joon Jung, Erlinda The, Gunjan Kukreja, Afarin Neishabouri, Azmi A. Ahmad, M. Zawwad Raza, Arvene Golbazi, Keshvad Hedayatyanfard, Lei Nie, Zhengxing Zhang, Lingyun Wang, Jiasheng Zhang, Mehran M. Sadeghi

**Affiliations:** 1 Section of Cardiovascular Medicine and Cardiovascular Research Center, Yale School of Medicine, New Haven, Connecticut, USA; 2 Section of Cardiology, VA Connecticut Healthcare System, West Haven, Connecticut, USA

**Keywords:** vascular endothelial growth factor, signaling, receptor regulation, protein motif, protein sequence

## Abstract

Signal sequences (SS) target newly synthesized proteins to the endoplasmic reticulum and, eventually, the plasma membrane. Discoidin, CUB, and LCCL domain containing 2 (DCBLD2) is a transmembrane protein with an unusually long SS that interacts with vascular endothelial growth factor receptor (VEGFR)-2 and promotes VEGF-induced endothelial cell signaling, proliferation and migration, as well as angiogenesis. How DCBLD2 interacts with VEGFR-2 to modulate VEGF signaling remains unclear. We generated various constructs containing different combinations of DCBLD2 domains, including its SS, and conducted co-immunoprecipitation and signaling studies in HEK 293T and endothelial cells to identify the smallest DCBLD2 unit that interacts with VEGFR-2. Other proteins containing this unit were identified, and their interactions with VEGFR-2 were evaluated. The DCBLD2 SS interacted with VEGFR-2 and promoted VEGF signaling. The smallest unit in the DCBLD2 SS that interacted with VEGFR-2 was the L5VL5 sequence. Even after the central valine was removed, the L10 sequence mimicked the DCBLD2 SS effect on VEGF-signaling, while shorter or longer multi-leucine sequences were less effective. We identified several other human proteins containing such uncleaved multi-leucine SS, including CD45-associated protein (CD45-AP) and BRICHOS domain containing 5 (BRICD5). Both these proteins could interact with VEGFR-2, and CD45-AP could modulate VEGF signaling in endothelial cells. In conclusion, a multi-leucine motif in SS can directly engage in protein-protein interactions and modulate VEGF signaling. These findings refine the traditional view of SS as merely targeting elements, emphasizing their role in cellular signaling and opening new avenues for research and therapy.

The targeting of newly synthesized proteins to the endoplasmic reticulum (ER) and, eventually, the plasma membrane of eucaryotic cells is mediated by signal sequences (SS). For type I transmembrane and secretory proteins, SS are typically 25 to 30 amino acid N-terminal extensions of the nascent proteins. During or after the completion of ER translocation, many SS are cleaved by signal peptidase to generate a signal peptide (SP), while others remain an integral part of the mature protein as signal anchor sequences (1, 2). Generally, cleaved SPs are rapidly degraded. However, some SPs and their fragments are retained and have specific, post-targeting functions on their own (3, 4). To date, however, most known examples of such SS post-targeting functions are of viral origin (5).

A typical SP has a tripartite structure with a hydrophobic, α-helical core (*h* region) surrounded by an N-terminal polar *n* region and a C-terminal *c* region that contains the signal peptidase cleavage site (1). Based on proteome analysis by machine-learning systems, it is suggested that exceptionally long SS may have a bipartite ‘NtraC’ domain structure with potentially functional N-terminal (N-domain, ‘N’) and C-terminal (C-domain, ‘C’) domains connected by a turn-rich linker area (transition area, ‘tra’) (6). One such protein featuring an exceptionally long SS is Discoidin, CUB and LCCL domain containing 2 (DCBLD2), whose 66 amino acid SS is predicted to fit the ‘NtraC’ model (7). The gene encoding DCBLD2, also known as endothelial and smooth muscle cell-derived neuropilin-like protein (ESDN) or CLCP1, was initially cloned from human coronary artery (8) and highly metastatic lung cancer cells (9) as a protein with the longest known secretory signal sequence among eukaryotes. DCBLD2 is highly conserved among vertebrates and closely resembles the structure of neuropilins (10, 11), major co-receptors for vascular endothelial cell growth factor (VEGF) receptors (VEGFRs), which play a critical role in angiogenesis. DCBLD2 is upregulated in human, rat, and mouse remodeling arteries (12, 13) and regulates VEGF-induced endothelial cell (EC) proliferation, migration, and signal transduction as well as developmental and adult angiogenesis (14).

Searching for DCBLD2 domain(s) involved in its interaction with VEGFR-2, we identified the DCBLD2 SS, specifically its ‘traC’ segment, as a key domain that interacts with VEGFR-2 and promotes VEGF/VEGFR-2 signaling in EC. The smallest unit in DCBLD2 SS that interacted with VEGFR-2 was the L5VL5 sequence, and even after the central valine is removed, the L10 sequence mimicked the DCBLD2 SS effect on VEGF-signaling. Finally, proteins containing a similar uncleaved multi-leucine SS could interact with VEGFR-2 and modulate VEGF signaling in endothelial cells. These findings establish a non-canonical function of SS in regulating growth factor signaling.

## Results

### The DCBLD2 SS traC subdomain is involved in DCBLD2 interaction with VEGFR-2

**Figure 1:**
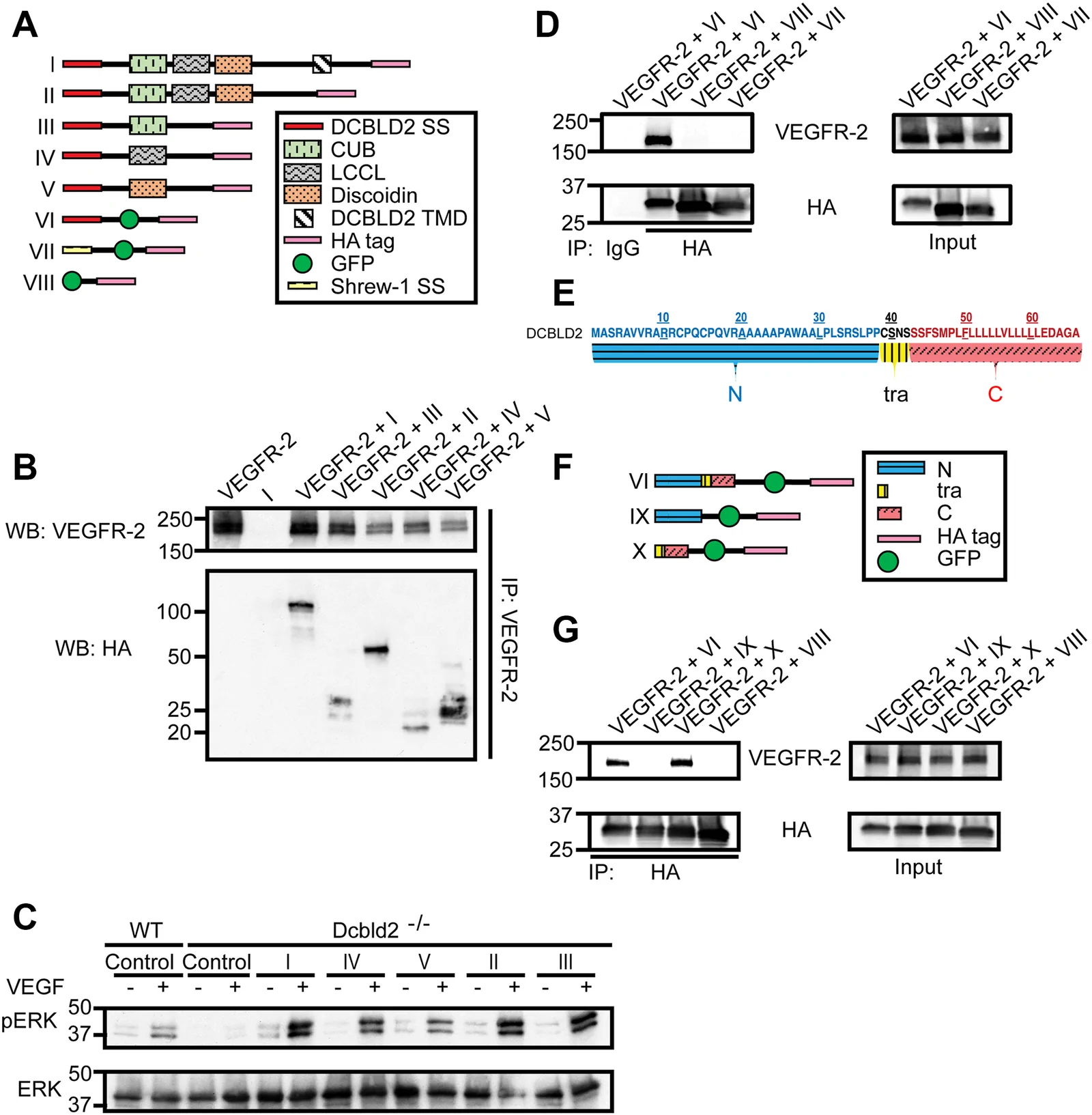
**The DCBLD2 SS traC subdomain is involved in DCBLD2 interaction with VEGFR-2.***A*, schematic representation of constructs I-VIII, each with various combinations of DCBLD2 domains tagged with HA (I-V), DCBLD2, or Shrew-1 SS fused with GFP and HA tags (VI, VII) or GFP-HA alone (VIII). *B*, co-immunoprecipitation of the products of constructs I-V with VEGFR-2 following transfection of each construct with VEGFR-2 (or transfection of construct I alone without VEGFR-2) in HEK 293T cells. VEGFR-2 immunoprecipitation is followed by Western blotting for HA and VEGFR-2. *C*, Western blot analysis of the effects of constructs I-V on VEGF-induced ERK phosphorylation following their transfection in *Dcbld2*^*−/−*^ murine lung endothelial cells transfected. *D*, co-immunoprecipitation of the products of constructs VI-VIII with VEGFR-2 following their transfection in HEK 293T cells. HA immunoprecipitation is followed by Western blotting for HA and VEGFR-2. *E*, NtraC model of DCBLD2 SS. *F*, schematic representation of constructs IX-XI encompassing different DCBLD2 SS subdomains fused with GFP and HA tags (IX-XI) or GFP-HA alone (VIII). *G*, co-immunoprecipitation of the products of constructs IX-XI with VEGFR-2 following their transfection in HEK 293T cells. HA immunoprecipitation is followed by Western blotting for HA and VEGFR-2. SS: signal sequence; TMD: transmembrane domain. HA: Hemagglutinin; GFP: green fluorescent protein; IP: immunoprecipitation; WB: Western blot; WT: wild type.

Distinct functional and structural units of DCBLD2 include an unusually long SS, which, in tandem with CUB, LCCL, and Discoidin domains, form the extracellular segment. A transmembrane domain and an intracellular segment comprise the rest of the molecule. To identify the key domain(s) of DCBLD2 that interact with VEGFR-2, we generated several plasmids expressing various combinations of different DCBLD2 domains linked to a C-terminal hemagglutinin (HA) tag (Fig. 1*A* I-V. Each construct expressed the appropriate size protein upon co-transfection with VEGFR-2 in HEK 293T cells (Fig. S1). Surprisingly, co-immunoprecipitation with an anti-VEGFR-2 antibody showed that all these products complex with VEGFR-2 (Fig. 1*B*). Furthermore, as reported previously (14), VEGF signaling was diminished in *Dcbld2*-deficient murine lung endothelial cells (MLEC), and all these DCBLD2 constructs enhanced VEGF-induced ERK phosphorylation upon transfection in *Dcbld2*^−/−^ MLEC (Fig. 1*C*). This led us to speculate that a common component of the constructs, *that is*, the SS, might be involved in its interaction with VEGFR-2 and enhancing VEGFR-2 signaling.

To test this possibility and to rule out HA as the VEGFR-2 interacting component, we constructed additional plasmids expressing DCBLD2 SS, Shrew-1 SS (another unusually long SS with a similar ‘NtraC’ domain structure (6)) or no SS in tandem with C-terminal green fluorescent protein (GFP)-HA tags (Fig. 1*A* VI-VIII). Upon co-transfection of these constructs with VEGFR-2 in HEK 293T cells and HA immunoprecipitation, VEGFR-2 could be pulled down by GFP-HA-tagged DCBLD2 SS, but not Shrew-1 SS or GFP-HA without any SS (Fig. 1*D*). In addition, despite a similar predicted size, the product of DCBLD2 SS-GFP-HA plasmid appeared larger on Western blots than the products of Shrew-1 SS-GFP-HA or GFP-HA constructs (Fig. 1*D*). This raised the possibility that the Shrew-1 SS but not the DCBLD2 SS is cleaved in the final product. Indeed, the SignalP 6.0 server, which can predict the location of the signal peptide cleavage site (15), suggested that, unlike Shrew-1, the N-terminal SS is not cleaved in DCBLD2 (Fig. S2). To confirm this experimentally, we transfected HEK 293T cells with a dually tagged DCBLD2 plasmid with N-terminal FLAG and C-terminal HA tags. Immunoprecipitation of the product with an anti-HA antibody followed by FLAG and DCBLD2 immunoblotting showed a single (noncleaved) product (Fig. S3). Replacing one or both alanine residues in the putative A-X-A signal peptidase cleavage site (16) of DCBLD2 with glycine led to similarly sized products, indicating that the DCBLD2 SS remains uncleaved (Fig. S3). Finally, VEGF-induced ERK phosphorylation in *Dcbld2*^*−/−*^ MLEC was enhanced upon transfection with the DCBLD2 SS-GFP-HA construct (Fig. S4). Taken together, these data suggest that the DCBLD2 SS remains an integral component of the mature DCBLD2 and is a key domain in its interaction with VEGFR-2, which modulates downstream VEGF signaling.

Based on the ‘NtraC’ model, the DCBLD2 SS 'N' subdomain contains 38 amino acid residues, and the 24 amino acid C-terminal residues constitute the ‘C’ subdomain, with those two separated by a short ‘tra’ subdomain (Fig. 1*E*). Hydrophobicity analysis by ProtScale (17) (Fig. S5) and the transmembrane helix prediction by DeepTMHMM (18) (Fig. S6) indicated significant differences between different DCBLD2 SS subdomains, with the ‘C’ subdomain adjacent to ‘tra’ predicted to be hydrophobic and transmembrane. To identify the subdomain of DCBLD2 SS that interacts with VEGFR-2, we synthesized additional constructs IX and X, expressing different DCBLD2 SS subdomains (Fig. 1*F*), which were co-transfected with VEGFR-2 into HEK 293T cells. Co-immunoprecipitation studies indicated that the ‘traC’ subdomain, but not the ‘N’ subdomain, is involved in the DCBLD2-VEGFR-2 interaction (Fig. 1*G*).

### A multi-leucine sequence can interact with VEGFR-2

**Figure 2:**
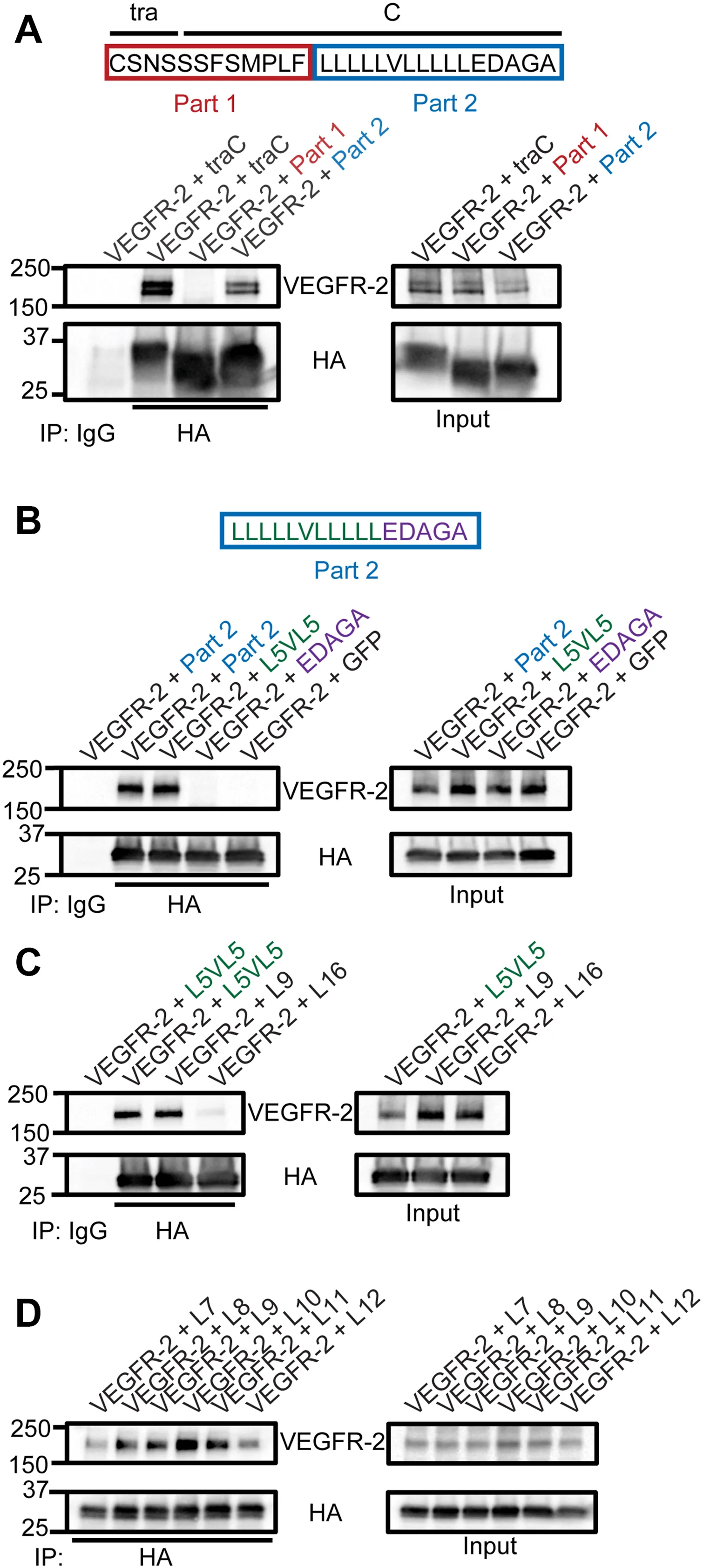
**A poly leucine decapeptide mimics the effect of DCBLD2 SS traC on VEGF signaling.***A*, co-immunoprecipitation of the products of HA-tagged traC Part 1 or Part 2 constructs with VEGFR-2 following their co-transfection in HEK 293T cells. HA immunoprecipitation is followed by western blotting for HA and VEGFR-2. *B*, co-immunoprecipitation of the products of HA-tagged traC Part 2 or its derivative constructs or a control HA-tagged GFP with VEGFR-2 following their co-transfection in HEK 293T cells. HA immunoprecipitation is followed by western blotting for HA and VEGFR-2. *C*, co-immunoprecipitation of the products of HA-tagged traC Part 2 L5VL5 sequence, or 9 or 16 multi-L-leucine constructs with VEGFR-2 following their co-transfection in HEK 293T cells. HA immunoprecipitation is followed by western blotting for HA and VEGFR-2. *D*, co-immunoprecipitation of the products of HA-tagged polyleucine constructs with VEGFR-2 following their co-transfection in HEK 293T cells. HA immunoprecipitation is followed by western blotting for HA and VEGFR-2.

Next, we sought to identify the smallest TraC sequence that interacts with VEGFR-2. The DCBLD2 SS traC was split into two parts, with an N-terminal part 1 (CSNSSSFSMPLF) and a C-terminal part 2 (LLLLLVLLLLLEDAGA) encompassing the multi-leucine sequence. Following co-transfection of plasmids expressing VEGFR-2 with GFP-HA-tagged part 1 or part 2 into HEK 293T cells, part 2, but not part 1, could be co-immunoprecipitated with VEGFR-2 (Fig. 2*A*). Part 2 was then split into two sub-parts: LLLLLVLLLLL (L5VL5) and EDAGA, each connected to a C-terminal GFP-HA tag. Following co-transfection with VEGFR-2 in HEK 293T cells, only the sequence L5VL5, and not EDAGA, could co-precipitate with VEGFR-2, indicating that this segment is involved in the DCBLD2 SS interactions with VEGFR-2 (Fig. 2*B*).

A comparison of the rat, mouse, and human DCBLD2 amino acid sequences showed that while the three proteins share a multi-L segment in their N-termini, the L5VL5 is replaced by L9 in the murine and rat proteins (Fig. S7). We, therefore, generated a set of plasmids that express a 9 or 16 leucine peptide connected to a C-terminal GFP-HA tag. Following co-transfection with VEGFR-2 in HEK 293T cells, co-immunoprecipitation studies showed that removing the valine in the L5VL5 sequence does not affect its interaction with VEGFR-2. Interestingly, however, elongating the polyleucine moiety to 16 amino acids hampered VEGFR-2 co-immunoprecipitation (Fig. 2*C*). Evaluation of additional constructs encompassing 7 to 12 leucine residues showed that the nine to 11-leucine sequences appear to have the strongest interaction with VEGFR-2, and this interaction diminishes as the number of leucine residues increases or decreases (Fig. 2*D*).

### L10-containing proteins interact with VEGFR-2 and regulate VEGF signaling

**Figure 3:**
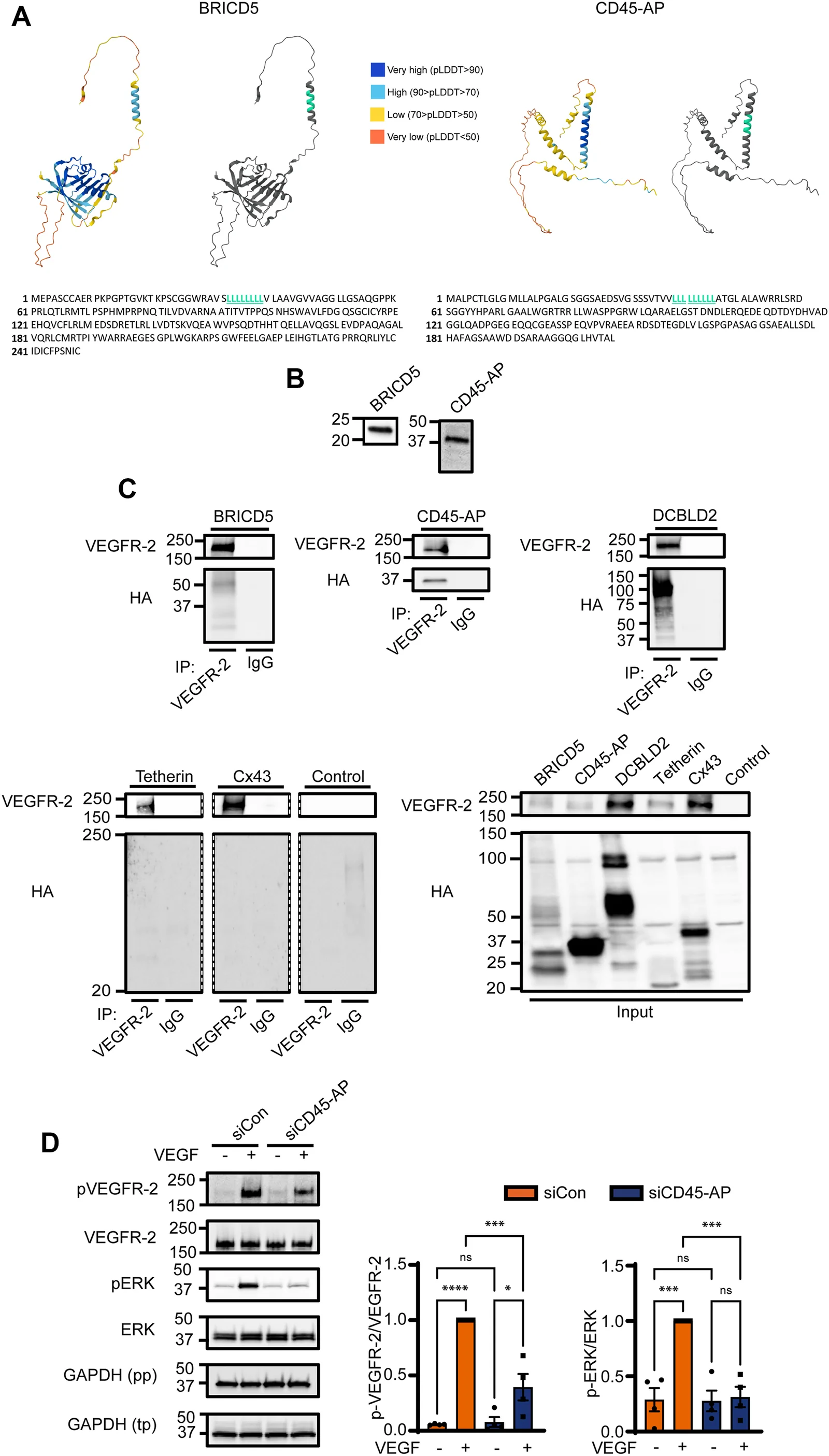
**BRICD5 and CD45-AP form complexes with VEGFR-2 and regulate VEGF signaling.***A*, predicted structure of BRICD5 (*left*) and CD45-AP (*right*) obtained from the AlphaFold protein structure database. The colors indicate the predicted Local Distance Difference Test (pLDDT) score, a measure of the residue level confidence in the 3D structure. The model highlights the multi-leucine sequence in *green*, and the corresponding amino acid sequence is highlighted below each model. *B*, Western blot analysis of BRICD5 and CD45-AP expression in endothelial cells. *C*, co-immunoprecipitation of HA-tagged CD45-AP and BRICD5 with VEGFR-2 following their co-transfection in HEK293T cells. VEGFR-2 immunoprecipitation is followed by western blotting for HA and VEGFR-2. DCBLD2 was used as positive control, whereas tetherin and connexin 43 (Cx43), which lack the multi-leucine sequences, co-transfected HEK293T cells and non-transfected HEK293T cells served as negative controls. *D*, Western blot analysis of VEGF-induced VEGFR-2 and ERK phosphorylation after siRNA-mediated knockdown of CD45-AP in HUVECs (n = 4 independent biological replicates). GAPDH was used as a loading control, with separate blots for total proteins (tp) and phosphorylated proteins (pp). ∗: *p* < 0.05, ∗∗∗: *p* < 0.001, ∗∗∗∗: *p* < 0.0001, ns = non-significant. Statistical significance was determined by one way ANOVA and Tukey’s multiple comparison *post hoc* test. Full ANOVA results are provided in Table S3.

L10's participation in VEGFR-2 interactions prompted us to investigate whether other proteins containing this sequence can interact with VEGFR-2 and regulate VEGF signaling. To address this, we performed a BLAST search of the L10 sequence against the human subset of UniProt's Swiss-Prot database and selected the top 50 hits with the highest alignment scores. We then analyzed these candidates with SignalP 6.0 to determine whether they were predicted to contain a cleavable signal peptide and narrowed our focus to proteins lacking a predicted cleavage site, ultimately identifying 9 such proteins. As proof of concept, we selected BRICHOS domain containing 5 (BRICD5) and protein tyrosine phosphatase receptor type C-associated protein (PTPRCAP or CD45-AP) for further evaluation (Fig. 3*A*). Given the paucity of information about these proteins, we first evaluated and confirmed the expression of both proteins in endothelial cells (Fig. 3*B*). Upon co-transfection with VEGFR-2 in HEK 293T cells, the HA-tagged BRICD5 and HA-tagged CD45-AP constructs expressed the appropriate-size proteins (Fig. 3*C*) and co-immunoprecipitation studies indicated that both proteins [but not control proteins lacking the L10 sequence, tetherin and connexin 43 (Cx43)], form a complex with VEGFR-2 (Fig. 3*C*). Finally, siRNA-mediated downregulation of CD45-AP diminished VEGF signaling in endothelial cells (Fig. 3*D*), indicating that like DCBLD2, CD45-AP can regulate VEGF signaling.

## Discussion

This study uncovered a new non-canonical function for the unusually long signal sequence of DCBLD2. DCBLD2 regulates developmental and adult angiogenesis by engaging in a macromolecular complex with VEGFR-2, which promotes VEGF signaling by dissociating VEGFR-2 from its negative regulators of downstream signaling, VE-cadherin and protein tyrosine phosphatases, PTP1 and TC-PTP (14). We identified the hydrophobic ‘traC’ subdomain of the DCBLD2 SS, specifically, its L5VL5 segment, as a key component of the DCBLD2 interaction with VEGFR-2. Notably, other human proteins containing a similar uncleaved multi-leucine SS, BRICD5 and CD45-AP, could interact with VEGFR-2, and CD45-AP could modulate VEGF signaling in endothelial cells.

Signal sequences are typically composed of 25 to 30 amino acids. Many are cleaved off the nascent protein by signal peptidase I as signal peptides. The latter is often rapidly degraded by signal peptide peptidase after directing the protein to the endoplasmic reticulum (19). However, in some cases, the signal peptide remains stable after cleavage and may be involved in additional post-targeting functions as transmembrane or cytosolic/luminal endoplasmic reticulum peptides (5, 20). Non-cleaved signal sequences remain an integral component of the mature protein as signal anchor sequences (2, 21, 22). Several characteristics of the signal sequence, including its length, hydrophobicity, and charge of the residues surrounding the transmembrane core, influence the topology of a signal anchor (23). Long signal sequences (>40 residues) are less common in eucaryotes and are speculated to have additional post-targeting functions (24). Many of these long signal sequences may have a modular, bipartite ‘NtraC’ domain structure, with the C-domain performing as a fully functional SP for endoplasmic reticulum targeting (6). The N domain, on the other hand, may have additional targeting functions. In the case of Shrew-1 and DCBLD2, it has been reported that the SS N domain may target the proteins to mitochondria (2, 7, 23). Our data are consistent with the prediction models and indicate that the DCBLD2 SS is not cleaved. Transmembrane domains are often engaged in protein-protein interactions and regulate the activity of the parent molecule. Similarly, the DCBLD2 SS, and more specifically its traC subdomain is involved in DCBLD2-VEGFR-2 interactions and modulates VEGF signaling.

We further determined that the smallest unit in the DCBLD2 SS capable of interacting with VEGFR-2 is the L5VL5 sequence. It is reported that most short multi-leucine transmembrane domains containing single amino acid substitutions can bind to and activate PDGFRβ or the erythropoietin receptor (25). Minor changes in the composition of these sequences (*e.g.*, changing the placement of a single side chain methyl group) can alter their partner selectivity (26, 27). Interestingly, even after removing the central valine, the L10 sequence could interact with VEGFR-2. However, shorter or longer multi-leucine sequences were less effective in this regard, suggesting that multi-leucine sequence length is a fundamental determinant of these interactions. Leucine repeats are common in signal sequences, especially in higher organisms (28) and their conservation in mammals points to specific functions. Consistent with this idea, we identified additional proteins containing multi-leucine sequences, such as BRICD5 and CD45-AP, that interact with VEGFR-2. These results underscore the broader functional importance of multi-leucine motifs, as further illustrated by the regulation of VEGFR-2 signaling by the DCBLD2 SS L5VL5. While we have shown that the DCBLD2 SS shows some specificity concerning its interactions with VEGFR-2 (compared to PDGFRβ), the possibility of its involvement in other signaling pathways not tested here cannot be ruled out.

VEGFR-2 is a primary receptor for VEGF, the prototypic pro-angiogenesis growth factor, in endothelial cells (29). Angiogenesis is a key process during development, growth, and adulthood. Inadequate neovessel formation is a major pathologic feature in ischemia-related cardiovascular diseases and contributes to poor wound healing. In other pathological settings, *for example*, diabetic retinopathy and malignancy, angiogenesis is improperly exaggerated. As such, there is considerable interest in regulators of the VEGF signaling pathway. The role of L5VL5 and L10 SS-containing proteins, along with their polyleucine-containing derivative peptides in related signaling pathways and cell types, will be the subject of future studies, which could also identify the specific VEGFR-2 sequences/domains that interact with L5VL5 and L10.

In conclusion, the L5VL5 and L10 containing signal sequences have a post-targeting role in interacting with and regulating VEGFR-2 signaling. Therefore, in addition to the potential of signal sequences as therapeutic targets through interference with co-translational translocation (30, 31, 32), this study highlights another aspect of the function of L5VL5 and L10-containing signal sequences: their role in protein-protein interactions, which could be targeted by agonists or antagonists to develop therapeutic agents.

## Experimental procedures

### Cell culture

HEK 293T cells (ATCC) were maintained in Dulbecco's Modified Eagle Medium (DMEM, Gibco) supplemented with 10% heat-inactivated fetal bovine serum (FBS, Sigma-Aldrich) and 1% penicillin/streptomycin (Gibco). For endothelial cell culture, primary human umbilical vein endothelial cells (HUVECs) were obtained from Yale Vascular Biology & Therapeutics Program Tissue Culture program and cultured on gelatin-coated cultureware with Medium 199 (Gibco) supplemented with 20% FBS, 1% endothelial cell growth supplement (ECGS), 2 mM L-glutamine, and 1% penicillin/streptomycin. MLECs were isolated from 4-week-old *Dcbld2*^*−/−*^ mice, under protocols approved by the Yale University Institutional Animal Care and Use. Mice were anesthetized and lungs harvested, rinsed in Hank's Balanced Salt Solution (HBSS, Sigma-Aldrich), cut into small pieces, and subjected to enzymatic digestion with a 1 mg/ml collagenase solution for 45 min. The tissue solution was then mechanically disrupted, passed through a 70 μm strainer, and the cells were collected by centrifugation at 350*g* for 5 min and resuspended in complete Medium 199. CD31-positive cells were separated using immunomagnetic separation with CD31-conjugated beads, then detached from the beads and cultured until 70 to 80% confluent. Finally, cells were subjected to another round of immunoselection with CD102-conjugated beads, following the same procedure and detachment steps. The purity of isolated MLECs was verified by flow cytometry analysis of CD31 expression. The antibody-conjugated magnetic beads were prepared according to the manufacturer’s (Thermo Fisher) instructions. A 0.25% trypsin-EDTA (Gibco) solution was used, following standard lab protocols, to pass adherent cells.

### Plasmid construction and transfection of plasmid DNA and siRNA

The DCBLD2-related plasmids used in this study are schematically shown in Figure 1, *A* and *F*. The genes encoding different protein fragments were cloned into a pCDNA3.0 plasmid between HindIII and KpnI restriction sites (9). The pCDNA3.4-CD45AP-HA (C terminal), pCDNA3.4-BRICD5-HA (C terminal), and pCDNA3-Flag-DCBLD2-HA (N terminal Flag, C terminal -HA) plasmids were generated by Quintara Bio. The pCDNA3-Cx 43-HA (C terminal HA) construct was from Addgene. All constructs were confirmed by PCR or DNA sequencing. Cells were transfected with plasmid DNA using Lipofectamine 3000 (Thermo Fisher) according to the manufacturer’s instructions. Twenty-four hours after transfection, cells were washed and cultured in DMEM supplemented with 10% FBS for another 24 h before use. The same Lipofectamine protocol was used for siRNA-mediated knockdown, and cells were maintained in M199 with 10% FBS 24 h post-transfection.

### Immunoblotting and immunoprecipitation

Cells were lysed on ice using ice-cold RIPA buffer (Thermo Fisher) supplemented with protease and phosphatase inhibitor cocktails (Roche). The lysates were centrifuged at 14,000*g* for 15 min at 4 °C, and the supernatant was collected. Protein concentrations were determined using BCA assays (Thermo Fisher). Equal amounts of protein were subjected to SDS-PAGE and transferred onto 0.2 μm PVDF membranes (Bio-Rad). Membranes were blocked for 1 h with 5% BSA (Sigma-Aldrich) in TBST (Cell Signaling). For immunoblotting, membranes were incubated overnight at 4 °C with primary antibodies and for 1 h at room temperature with secondary antibodies. The blots were developed using an ECL substrate (Thermo Fisher) and imaged using a ChemiDoc MP imaging system (Bio-Rad). For quantification, band intensities were measured using Image Lab software (Bio-Rad). Target protein signal was normalized to GAPDH and reported relative to controls. For co-immunoprecipitation studies, Dynabeads Protein A Immunoprecipitation Kit (Invitrogen) was used. All steps were carried out according to the manufacturer's protocol. After completing the protocol, beads were removed with a magnet, and the resultant supernatant was collected for analysis.

### Statistical analysis

Results are expressed as the mean ± standard deviation. Statistical analysis was performed using GraphPad Prism 10. Data were checked for normality with the Shapiro-Wilk test. Statistical significance was determined by a two-way analysis of variance (ANOVA), followed by Tukey’s *post hoc* test for multiple groups. A *p*-value < 0.05 was considered significant.

## Data availability

All data are available in the main text or the supplementary materials.

## Supporting information

This article contains supporting information.

## Conflict of interest

The authors declare the following financial interests/personal relationships which may be considered as potential competing interests:

J.-J. J: Yale patent application: Peptides for promoting neovascularization and wound healing and methods using the same, Ownership and income, Arvinas, Inc.

M. M. S: Yale patent application: Peptides for promoting neovascularization and wound healing and methods using the same, Spouse employee of Boehringer Ingelheim.
