# Supplementary material for: A signal sequence multi-leucine motif regulates VEGF receptor signaling
Source: J Biol Chem. 2026 Aug 7;302(10):113406. doi: 10.1016/j.jbc.2026.113406 (PMC13570295; doi:10.1016/j.jbc.2026.113406)
Supplement: Supplementary file 1 [file mmc1.pdf]

A signal sequence multi-leucine motif regulates VEGF receptor signaling

Mean Ghim[1,2]†, Linyan Wei[1,2]†, Jae-Joon Jung[1,2], Erlinda The[1,2], Gunjan Kukreja[1,2], Afarin Neishabouri[1,2], Azmi A. Ahmad[1,2], M. Zawwad Raza[1,2], Arvene Golbazi[1,2], Keshvad Hedayatyanfard[1,2], Lei Nie[1,2], Zhengxing Zhang[1,2], Lingyun Wang[1,2], Jiasheng Zhang[1,2], Mehran M. Sadeghi[1,2]*

[1] Section of Cardiovascular Medicine and Cardiovascular Research Center, Yale School of Medicine; New Haven, CT, USA.

[2] VA Connecticut Healthcare System; West Haven, CT, USA.

†These authors contributed equally to this work

SUPPLEMENTAL FIGURES (S1-S7) and TABLES (S1-S3)

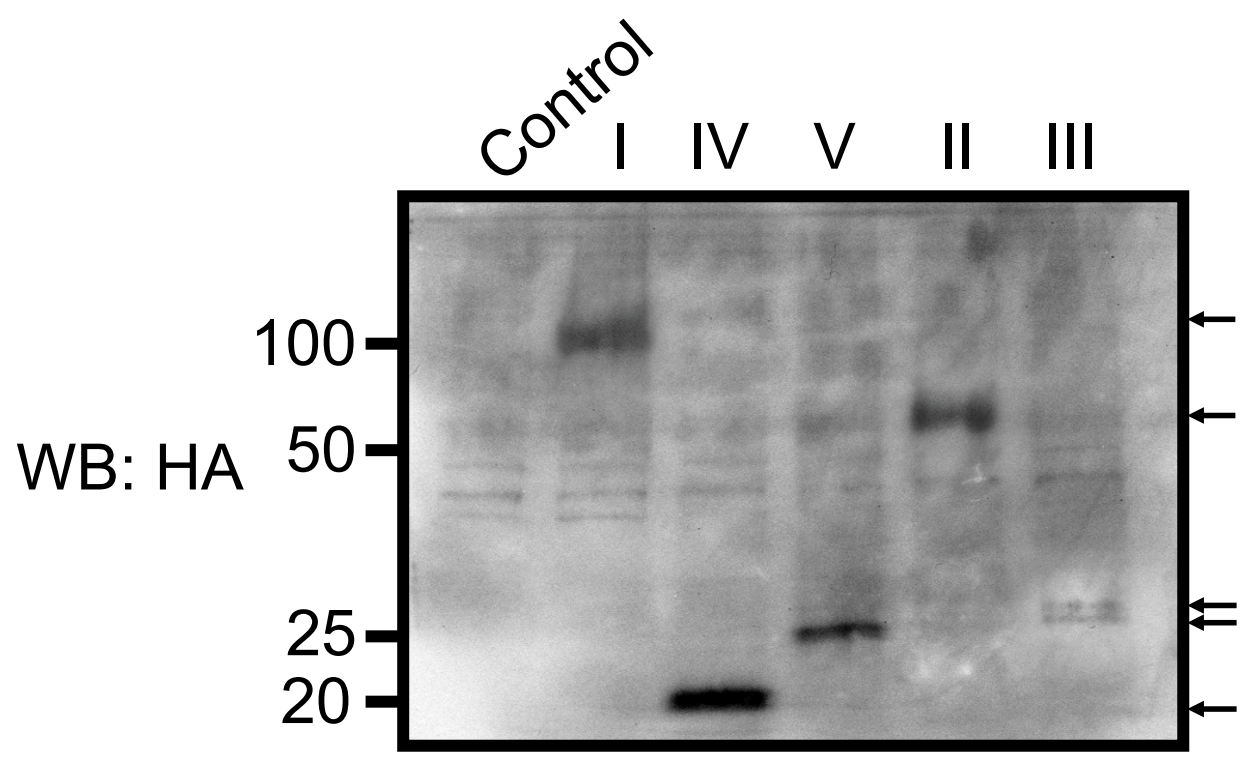

Fig. S1. Expression of DCBLD2 constructs in HEK 293T cells. Western blot of protein extracts from HEK 293T cells transfected with DCBLD2 constructs I-V, showing expression of appropriate size products.

DCBLD2

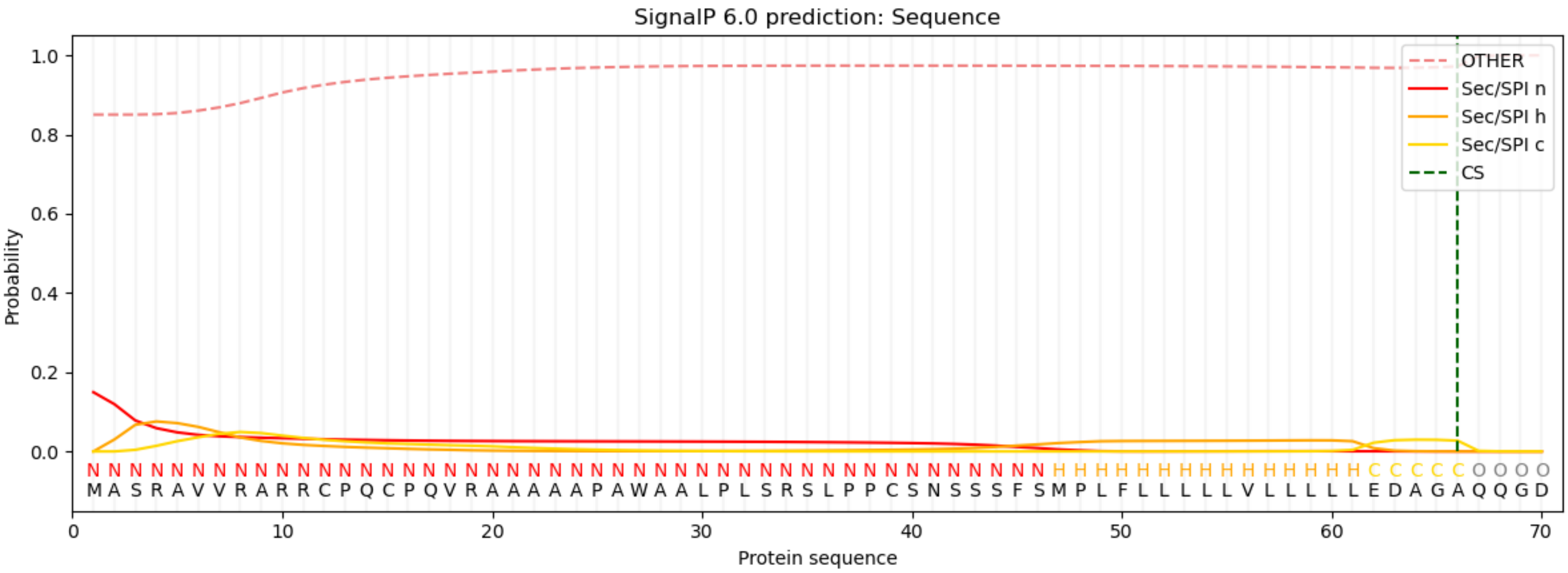

Sequence
Prediction: Other

Cleavage site between pos. 66 and 67.

Probability 0.027255

Shrew-1

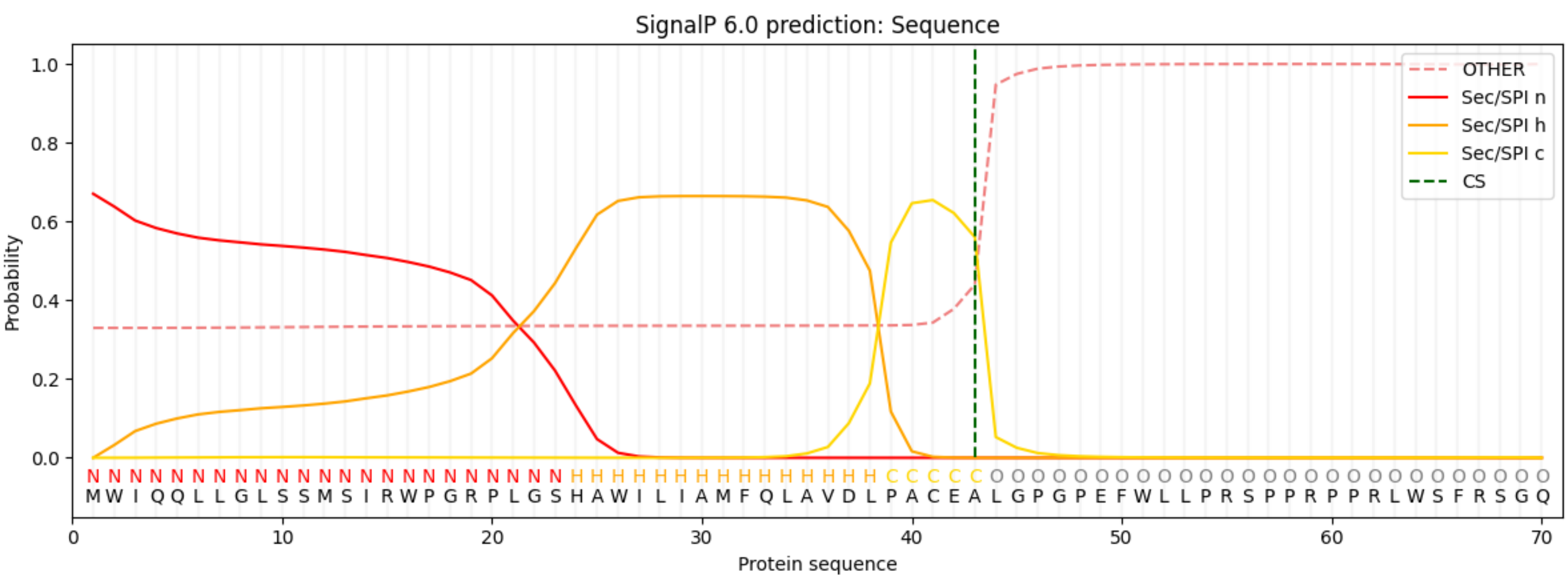

Sequence
Prediction: Signal Peptide (Sec/SPI)

Cleavage site between pos. 43 and 44.

Probability 0.560356

Fig. S2. Signal sequence cleavage prediction.
SignalP 6.0 prediction of DCBLD2 and SHREW1 signal sequence cleavage sites, showing the low probability of DCBLD2 signal sequence cleavage.

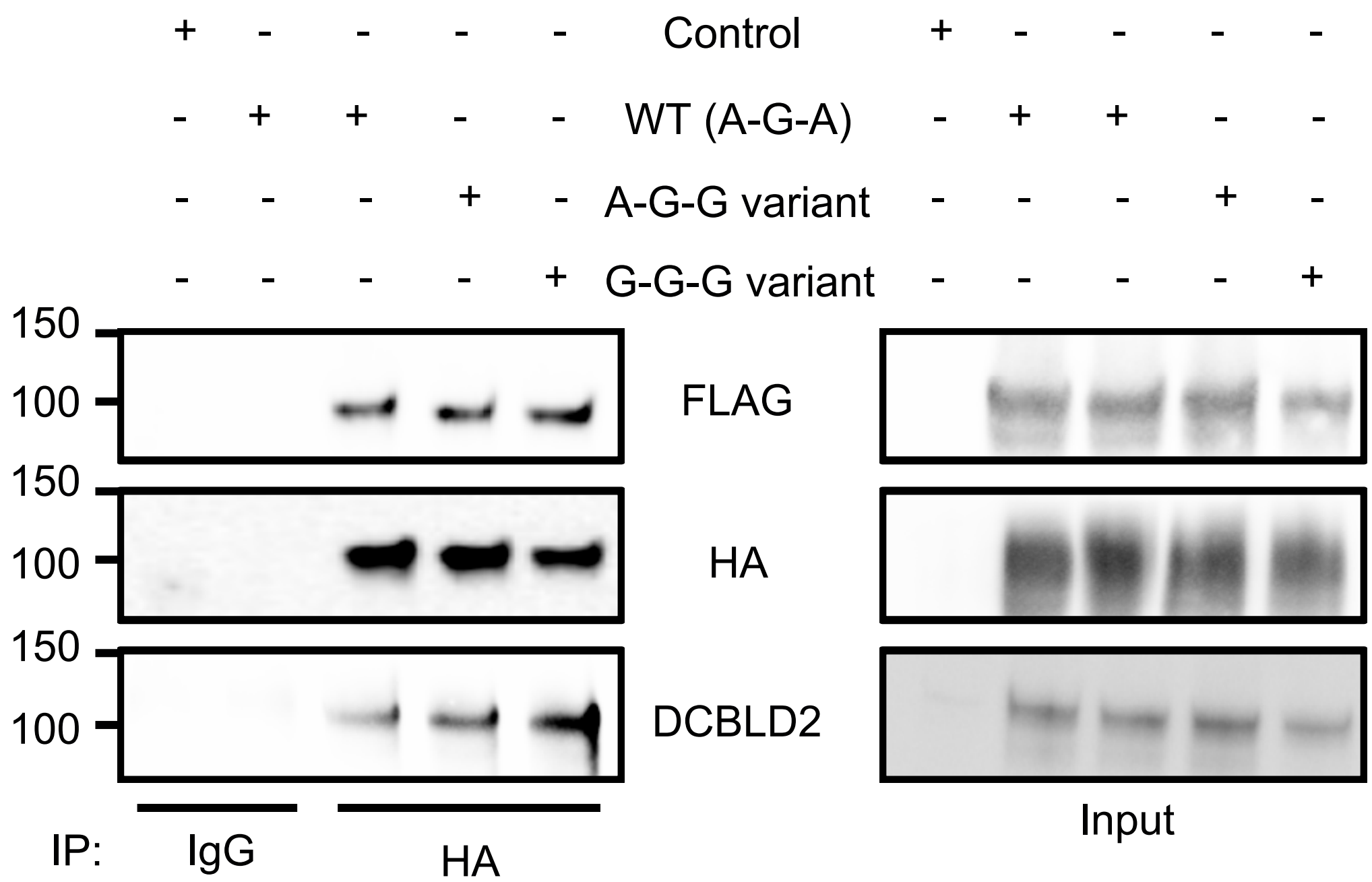

Fig. S3. DCBLD2 signal sequence cleavage determination.
HA immunoprecipitation of dually (N-terminal FLAG and C-terminal HA) tagged DCBLD2 and its mutant variants, where one or both alanine residues in the putative A-X-A signal peptidase cleavage site of DCBLD2 are replaced with glycine, transfected in HEK293T cells followed by FLAG, HA and DCBLD2 immunoblotting.

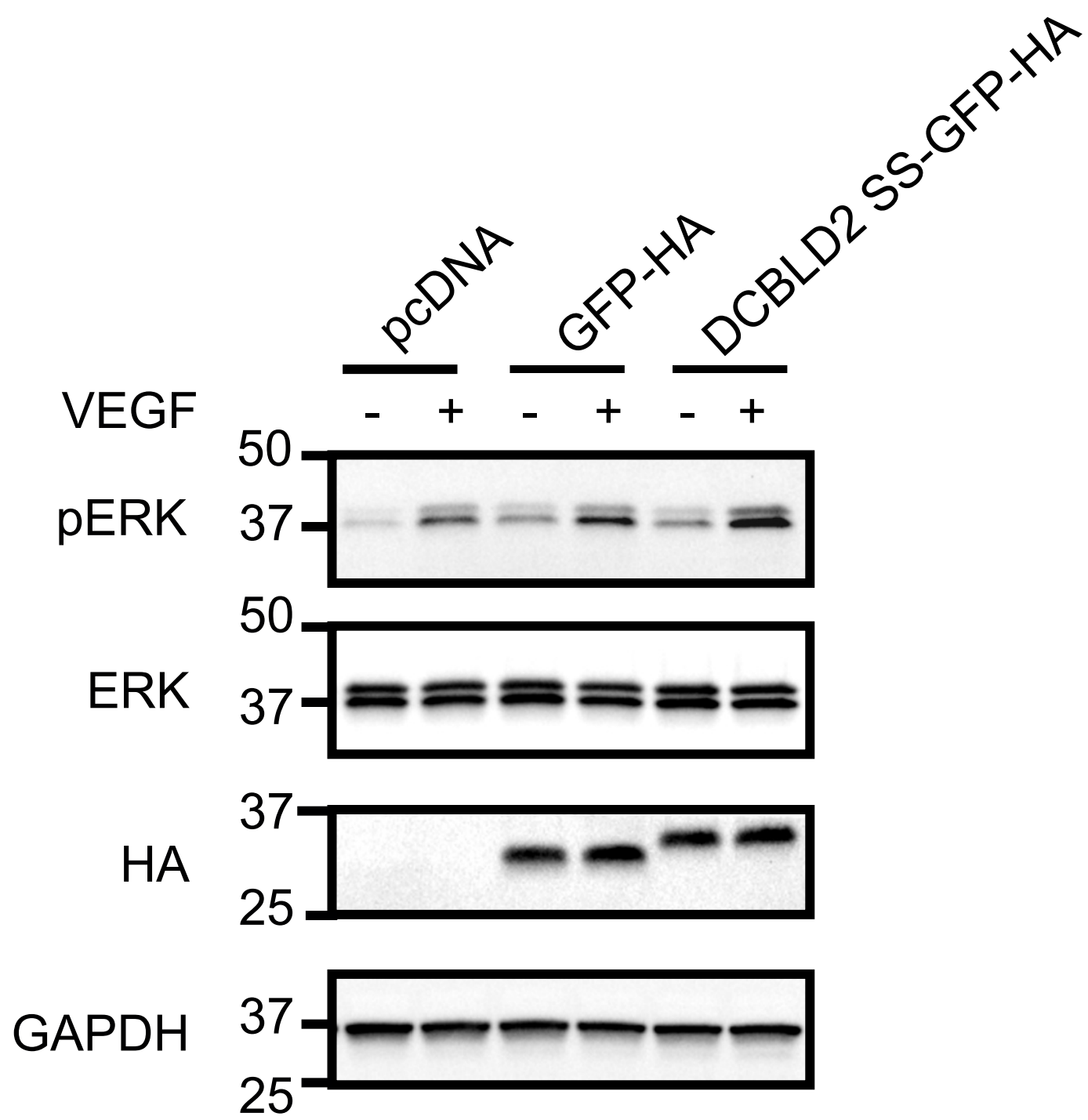

Fig. S4. Western blot analysis of VEGF-induced ERK phosphorylation in Dcbld2$^{-/-}$ murine endothelial cells transfected with DCBLD2-SS-GFP-HA or GFP-HA constructs.

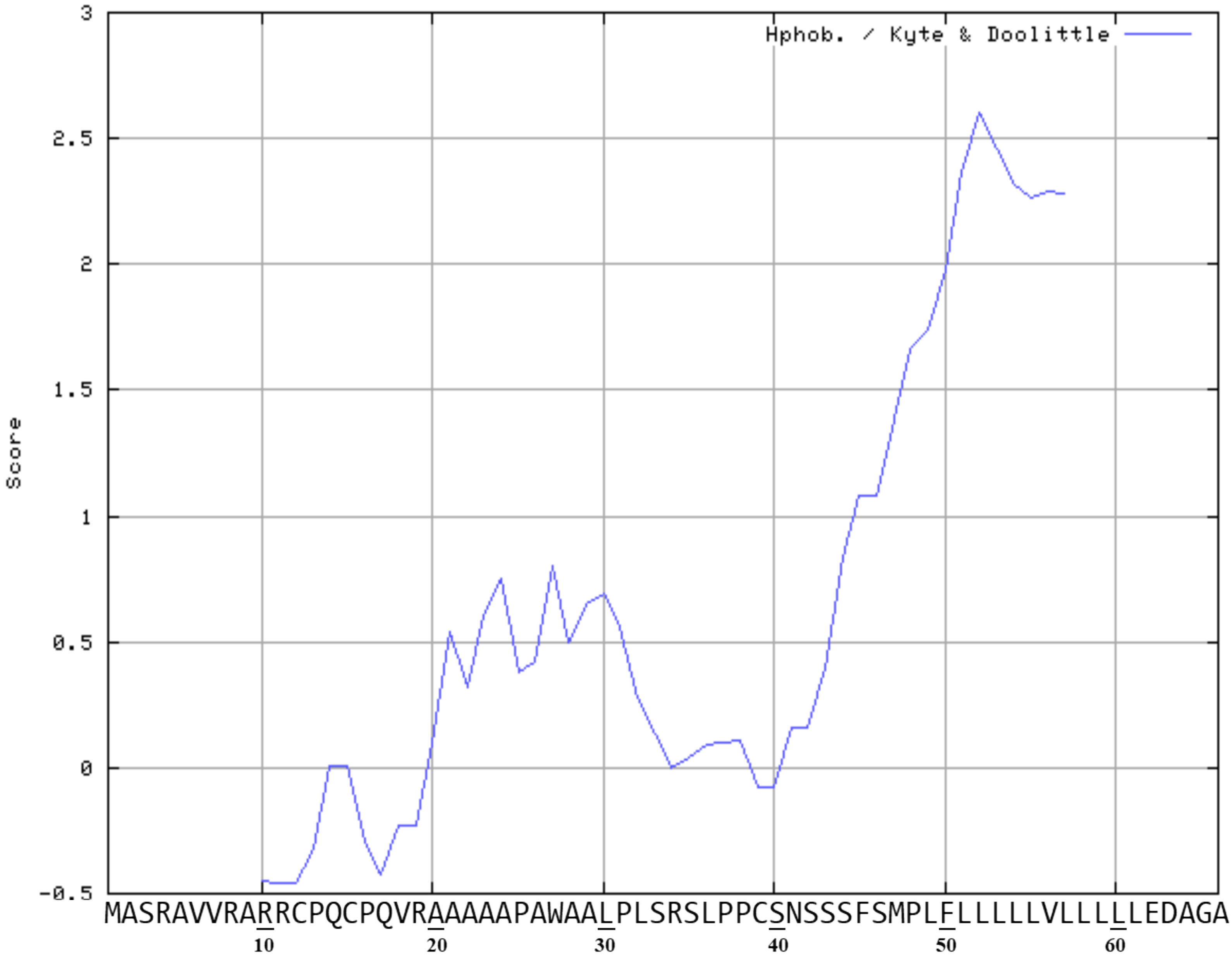

Fig. S5. Hydrophobicity analysis of the DCBLD2 signal sequence subdomains by ProtScale.

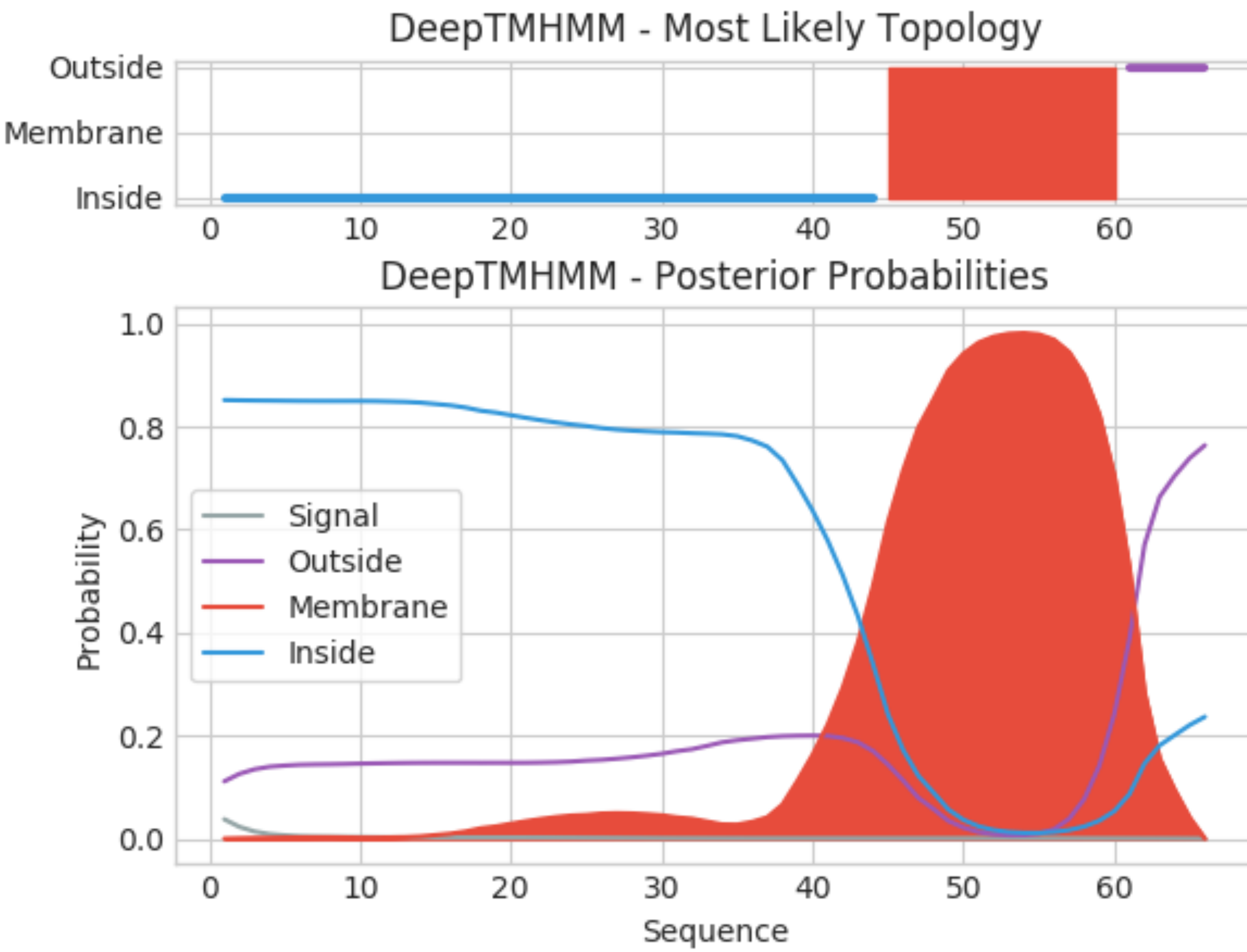

Fig. S6. DeepTMHMM prediction of transmembrane regions within the DCBLD2 signal sequence.

```
Rat    1    MASRAPLRAARSPQDPGGRAAPAATGRAPLPSAGWCPLPPGRNSSSRPR-----LLLLLLLLLPDAGAQKGDGCGHTVLG   75
Mouse  1    MASRAPLRAARSPQGPGGPAAPAATGRAALPSAGCCPLPPGRNSSSRPR-----LLLLLLLLLQDAGGQQGDGCGHTVLG   75
Human  1    MASRAVVRARRCPQCPQVRAAAAAPAWAALPLSR--SLPPCSNSSSFSMplfllLLLVLLLLLEDAGAQQGDGCGHTVLG   78

       76   PESGTLTSINYPHTYPNSTVCKWEIRVKTGERIRIKFGDFDIEDSDYCHLNYLKIFNGIGVSRTEIGKYCGLGLQMNQSI   155
       76   PESGTLTSINYPHTYPNSTVCEWEIRVRTGERIRIKFGDFDIEDSDYCHLNYLKIFNGIGVSRTEIGKYCGLGLQMNQSI   155
       79   PESGTLTSINYPQTYPNSTVCEWEIRVKMGERVRIKFGDFDIEDSDSCHFNYLRIYNGIGVSRTEIGKYCGLGLQMNHSI   158

       156  ESKGSEITVLFMSGIHASGRGFLASYSVIDKQDLITCLDTVSNFLEPEFSKYCPAGCLLPFAEISGTIPHGYRDSSPLCM   235
       156  ESKGSEVTVLFMSGTHAAGRGFLASYSVIDKEDLITCLDTVSNFLEPEFSKYCPAGCLLPFAEISGTIPHGYRDSSPLCM   235
       159  ESKGNEITLLFMSGIHVSGRGFLASYSVIDKQDLITCLDTASNFLEPEFSKYCPAGCLLPFAEISGTIPHGYRDSSPLCM   238

       236  AGIHAGVVSDVLGGQISVVISKGTPYYESSLANNVTSMVGYLSTSLFTFKTSGCYGTLGMESGVIADPQITASSVLEWTD   315
       236  AGIHAGVVSNVLGGQISIVISKGTPYYESSLANNVTSTVGYLSASLFTFKTSGCYGTLGMESGVIADPQITASSALEWTD   315
       239  AGVHAGVVSNTLGGQISVVISKGIPYYESSLANNVTSVVGHLSTSLFTFKTSGCYGTLGMESGVIADPQITASSVLEWTD   318

       316  HMGQENSWKPEKARLRKPGPPWAAFATDEHQWLQIDLNKEKKITGIVTTGSTLIEHNYYVSAYRVLYSDDGQKWTVYREP   395
       316  HMGQENSWTAEKARLRKPGPPWAAFATDEHQWLQIDLNKEKKITGIVTTGSTMIEHSYYVSAYRVLYSDDGQRWTVYREP   395
       319  HTGQENSWKPKKARLKKPGPPWAAFATDEYQWLQIDLNKEKKITGIITTGSTMVEHNYYVSAYRILYSDDGQKWTVYREP   398

       396  GAAQDKIFQGNKDYHKDVRNNFLPPIIARFIRVNPVQWQQKIAMKVELLGCQFTLKGRLPKLTQPPPPRNSNNLKNTTVH   475
       396  GVDQDKIFQGNKDYHKDVRNNFLPPIIARFIRVNPVQWQQKIAMKVELLGCQFTLKGRLPKLT--PPPRNGNNLRNTTAR   473
       399  GVEQDKIFQGNKDYHQDVRNNFLPPIIARFIRVNPTQWQQKIAMKMELLGCQFIPKGRPPKLTQPPPPRNSNDLKNTTAP   478

       476  PKLG--RAPKFTQALQPRSRNDLPLLPAQTTATPDVKNTTVTPSVTKDVALAAVLVPVLVMALTTLILILVCAWHWRNRK   553
       474  PKLGKGRAPKFTQVLQPRSRNELPVQPAETTTTPDIKNTTVTPSVTKDVALAAVLVPVLVMALTTLILILVCAWHWRNRK   553
       479  PKIAKGRAPKFTQPLQPRSSNEFPAQTEQTTASPDIRNTTVTPNVTKDVALAAVLVPVLVMVLTTLILILVCAWHWRNRK   558

       554  KKAEGTYDLPHWDRAGWWKGVKQLLPAKSVEHEETPVRYSNSEVSHLSPREVTTVLQADSAEYAQPLVGGIVGTLHQRST   633
       554  KKTEGAYDLPHWDRAGWWKGMKQLLPAKSVDHEETPVRYSTSEVSHLSAREVTTVLQADSAEYAQPLVGGIVGTLHQRST   633
       559  KKTEGTYDLPYWDRAGWWKGMKQFLPAKAVDHEETPVRYSSSEVNHLSPREVTTVLQADSAEYAQPLVGGIVGTLHQRST   638

       634  FKPEEGKEASYADLDPYNAPVQEVYHAYAEPLPVTGPEYATPIVMDMSGHSTASVGLPSTSTFRTAGNQPPALVGTYNTL   713
       634  FKPEEGKEAGYADLDPYNSPMQEVYHAYAEPLPVTGPEYATPIVMDMSGHPTASVGLPSTSTFKTAGTQPHALVGTYNTL   713
       639  FKPEEGKEAGYADLDPYNSPGQEVYHAYAEPLPITGPEYATPIIMDMSGHPTTSVGQPSTSTFKATGNQPPPLVGTYNTL   718

       714  LSRTDSCSSGQAQYDTPKGGKPAA-APEELVYQVPQSTQEASGAGRDEKFDAFKETL   769
       714  LSRTDSCSSGQAQYDTPKGGKSAA-TPEELVYQVPQSTQELSGAGRDEKFDAFKEIL   769
       719  LSRTDSCSSAQAQYDTPKAGKPGLpAPDELVYQVPQSTQEVSGAGRDGECDVFKEIL   775
```

Fig. S7. Alignment of rat, mouse, and human DCBLD2 protein sequences. The mouse and rat sequences contain a L9 sequence instead of the L5VL5 sequence in human DCBLD2.

Table S1. Materials and reagents used in the study

Materials and reagents	Supplier	Catalog number
Lipofectamine 3000	Thermo Fisher	L3000001
RIPA buffer	Thermo Fisher	89901
Protease inhibitor	Roche Applied Sciences	11873580001
Phosphatase inhibitor	Roche Applied Sciences	4906837001
BCA assays	Thermo Fisher	23225
PVDF membranes	Bio-Rad	1620174
BSA	Sigma Aldrich	A9647
TBST	Cell Signaling Technology	9997S
Dynabeads Protein A Immunoprecipitation Kit	Invitrogen	10006D
ECL substrate	Thermo Fisher	1863096
Recombinant human VEGF	R&D Systems	293-VE
Dulbecco's Modified Eagle Medium	Gibco	11965-092
Fetal bovine serum	Sigma Aldrich	12306C
Penicillin/streptomycin	Gibco	15140-122
Medium 199	Gibco	11150-059
DCBLD2 siRNA	Horizon	L-016714-00-0005
Control siRNA	Horizon	D-001810-01-05
Trypsin-EDTA	Thermo Fisher	25200056

Table S2. List of antibodies used in the study

Antibodies (dilutions used for WB)	Supplier	Catalog number
phospho–VEGFR-2 (Tyr1175) (1:1000)	Cell Signaling Technology	2478S
VEGFR-2 (1:1000)	Cell Signaling Technology	2479S
phospho-ERK 1/2 (Thr202/Tyr204) (1:1000)	Cell Signaling Technology	9101S
ERK 1/2 (1:2000)	Cell Signaling Technology	9102S
DCBLD2 (1:1000)	Cell Signaling Technology	4804S
BRICD5 (1:1000)	Thermo Fisher	PA5-66590
CD45-AP (1:1000)	Thermo Fisher	PA5-60487
GAPDH (1:5000)	Cell Signaling Technology	2118L
HA-Tag (1:1000)	Cell Signaling Technology	3724S
His-Tag (1:1000)	R&D Systems	MAB050
FLAG-Tag (1:1000)	Cell Signaling Technology	2368S
Rabbit-IgG (HRP-linked, 1:5000)	Cell Signaling Technology	7074S
Mouse-IgG (HRP-linked, 1:5000)	Cell Signaling Technology	7076S

Table S3. ANOVA statistics for Figure 3D

ANOVA summary					
F	17.83				
P value	0.0001				
P value summary	***				
Significant diff. among means (P < 0.05)?	Yes				
R squared	0.8168				
Brown-Forsythe test					
F (DFn, DFd)	2.854 (3, 12)				
P value	0.0817				
P value summary	ns				
Are SDs significantly different (P < 0.05)?	No				
ANOVA table	SS	DF	MS	F (DFn, DFd)	P value
Treatment (between columns)	1.504	3	0.5014	F (3, 12) = 17.83	P=0.0001
Residual (within columns)	0.3374	12	0.02812		
Total	1.842	15			
Data summary					
Number of treatments (columns)	4				
Number of values (total)	16				

Number of families	1							
Number of comparisons per family	6							
Alpha	0.05							
Tukey's multiple comparisons test	Mean diff.	95.00% CI of d	Below threshol	Summary	Adjusted P Value			
siCon vs. siCon+V	-0.7121	-1.064 to -0.36	Yes	***	0.0003	A-B		
siCon vs. siCD45-AP	0.01068	-0.3413 to 0.36	No	ns	0.9997	A-C		
siCon vs. siCD45-AP +V	-0.02461	-0.3766 to 0.32	No	ns	0.9967	A-D		
siCon+V vs. siCD45-AP	0.7228	0.3708 to 1.07	Yes	***	0.0003	B-C		
siCon+V vs. siCD45-AP +V	0.6875	0.3355 to 1.04	Yes	***	0.0004	B-D		
siCD45-AP vs. siCD45-AP +V	-0.03529	-0.3873 to 0.31	No	ns	0.9904	C-D		
Test details	Mean 1	Mean 2	Mean diff.	SE of diff.	n1	n2	q	DF
siCon vs. siCon+V	0.2879	1	-0.7121	0.1186	4	4	8.494	12
siCon vs. siCD45-AP	0.2879	0.2772	0.01068	0.1186	4	4	0.1274	12
siCon vs. siCD45-AP +V	0.2879	0.3125	-0.02461	0.1186	4	4	0.2935	12
siCon+V vs. siCD45-AP	1	0.2772	0.7228	0.1186	4	4	8.621	12
siCon+V vs. siCD45-AP +V	1	0.3125	0.6875	0.1186	4	4	8.2	12
siCD45-AP vs. siCD45-AP +V	0.2772	0.3125	-0.03529	0.1186	4	4	0.4209	12
Compact letter display								
siCon+V	A							
siCD45-AP +V	B							
siCon	B							
siCD45-AP	B							
